# Supplementary material for: Cancer-cell-secreted miR-204-5p induces leptin signalling pathway in white adipose tissue to promote cancer-associated cachexia
Source: Nat Commun. 2023 Aug 24;14:5179. doi: 10.1038/s41467-023-40571-9 (PMC10449837; doi:10.1038/s41467-023-40571-9)
Supplement: Supplementary file 1 — Supplementary Information [file 41467_2023_40571_MOESM1_ESM.pdf]

Supplementary information

Cancer-cell-secreted miR-204-5p induces leptin signaling pathway in white adipose tissue to promote cancer-associated cachexia

Yong Hu[1,2,5], Liu Liu[1,5], Yong Chen[1], Xiaohui Zhang[1], Haifeng Zhou[1], Sheng Hu[1], Xu Li[1], Meixin Li[1], Juanjuan Li[3], Siyuan Cheng[2], Yong Liu[1,4], Yancheng Xu[2]* and Wei Yan[1]*

Supplementary information includes nine Supplementary Figures and four Supplementary Tables.

Supplementary Figure 1. Cancer-derived sEVs induces white adipose tissue fat loss.

Supplementary Figure 2. Cancer cell-secreted miR-204 promotes hypoxia signaling by targeting *Vhl*.

Supplementary Figure 3. Cancer-secreted miR-204 induces leptin signaling pathway in white adipose tissue.

Supplementary Figure 4. Leptin receptor deficiency blocks miR-204 mediated white adipose tissue browning.

Supplementary Figure 5. Circulating miR-204 enhances phosphatidylethanolamines production to promote cancer-associated cachexia.

Supplementary Figure 6. Exogenous VHL expression suppresses white adipose tissue browning.

Supplementary Figure 7. Exosomal miR-204 promotes thermogenesis and lipolysis in LLC-xenografted tumour mice.

Supplementary Figure 8. sEV characterization and the effect of miR-204 sEVs on the tumour metastatic.

Supplementary Figure 9. Nanoflow cytometry gating strategy for MDA-MB-231 sEVs and MCF-10A sEVs.

Supplementary Table 1. Predicted miRNA regulators of *VHL*.

Supplementary Table 2. Oligonucleotide primers for qRT-PCR analysis.

Supplementary Table 3. Antibodies used in this study.

Supplementary Table 4. Clinical information included in this study.

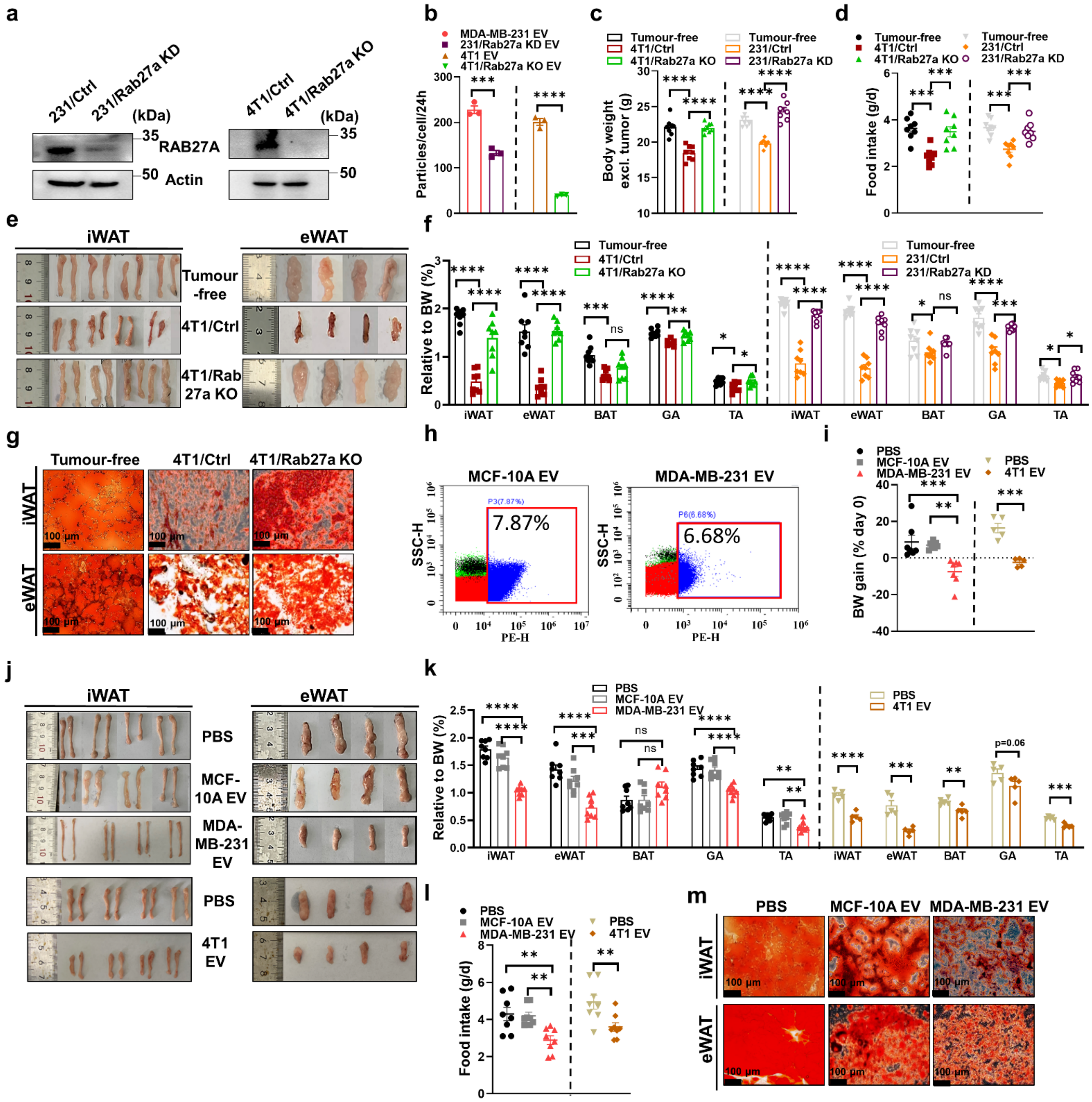

Supplementary Figure 1. Cancer-derived sEVs induces white adipose tissue fat loss.
(a) RAB27A level in MDA-MB-231/Rab27a KD cells and 4T1/Rab27a KO cells. **(b)** The particles of 231/Rab27a KD sEVs and 4T1/Rab27a KO sEVs (n=3 biologically independent samples per group). **(c)** Body weight (calculated by subtracting tumour weight from the total weight) of mice bearing indicated tumours (n=8 mice for Balb/c mice; n=7 mice for NSG mice), results of two independent experiments were combined). For the body weight changes of BALB/c mice (n=7 mice per group): 11.77 ± 1.63% (tumour-free group), -6.41 ± 1.36% (4T1/Ctrl group) and 12.19 ± 0.48% (4T1/Rab27a KO group); For the body weight change of NSG mice (n=5 per group): 7.05 ± 1.16% (tumour-free group), -7.57 ± 1.38% (231/Ctrl group) and 9.47 ± 3.52% (231/Rab27a KD group). **(d)** Daily food intake of mice in indicated groups (n=8 mice per group). **(e)** Representative adipose tissue (iWAT, eWAT) images in tumour group mice (n=4 mice in each group). **(f)** Relative tissues weight normalized to BW (body weight). n=8 mice in each group. **(g)** Oil Red O staining in adipose tissues of tumour group mice. Scale bar, 100 μm. **(h)** Nanoflow cytometry showing PE conjugated

CD9 expression on the sEVs membranes. **(i)** Body weight gain after 5 weeks sEV injection. n=7 mice for each group on the left; n=5 mice for each group on the right. **(j)** Representative adipose tissue (iWAT, eWAT) images in sEVs group mice (n=4 mice in each group). **(k)** Relative tissues weight (normalized to body weight). n=8 mice for each group on the left; n=5 mice for each group on the right. **(l)** Daily food intake of mice in indicated groups (n=8 mice per group). **(m)** Oil Red O staining in adipose tissues of indicated groups of mice. Scale bar, 100 μm. Oil Red O staining experiments were repeated three times independently with similar results obtained. Data are presented as mean ± s.e.m; *P<0.05, **P<0.01, ***P<0.001, ****P<0.0001, ns: not significant. One-way ANOVA followed by Dunnett multiple comparison test was used for c, d, f, i, k, l. Unpaired two-tailed t-test was used for b. Source data and exact *P* value are provided as a Source data file.

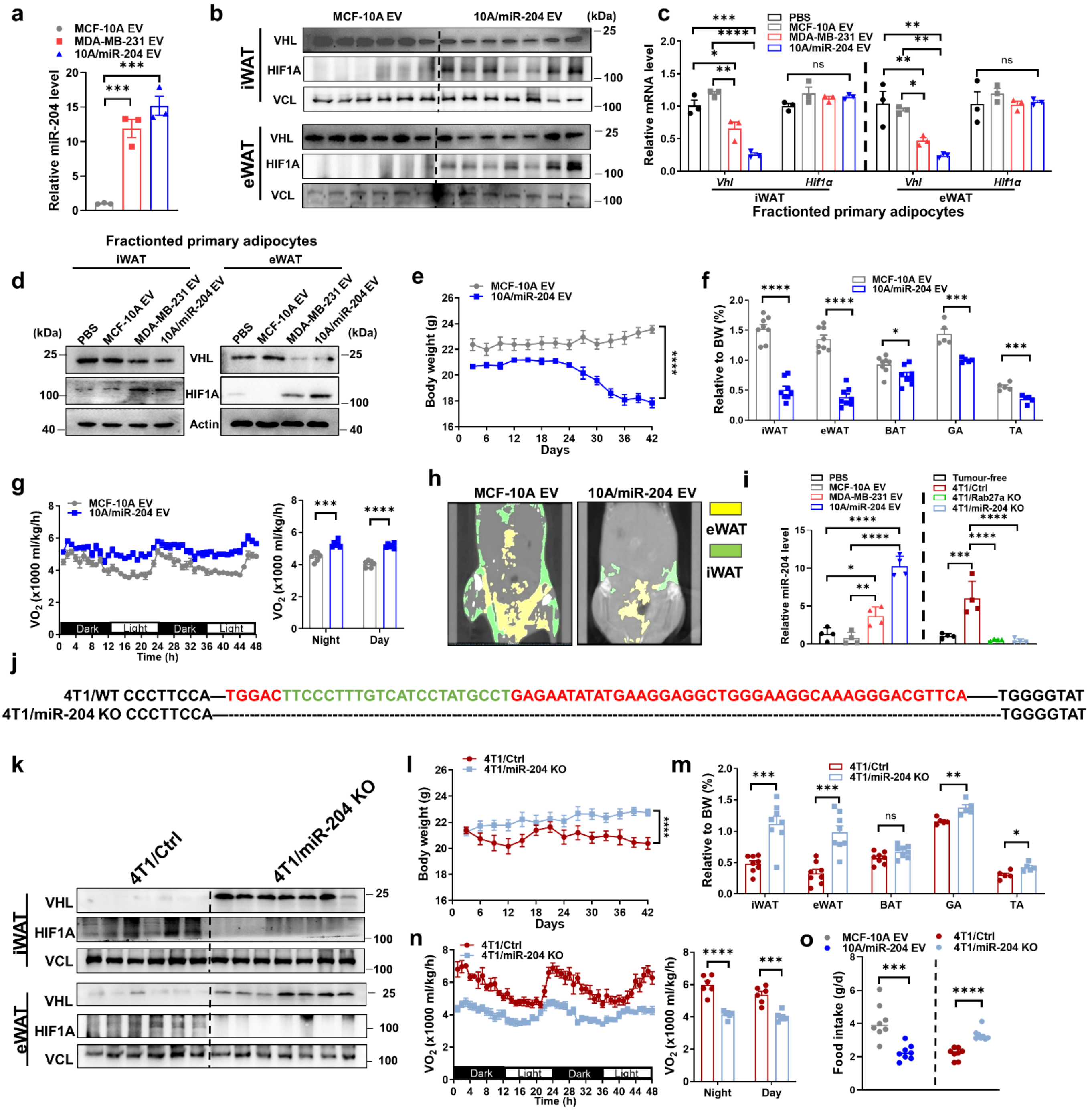

Supplementary Figure 2. Cancer cell-secreted miR-204 promotes hypoxia signaling by targeting Vhl. **(a)** The expression level of miR-204 in indicated sEVs (n=3 biologically independent samples per group). **(b)** Western blots showing indicated proteins levels in iWAT and eWAT from sEVs injected NSG mice in indicated groups. (n=6 mice for MCF-10A sEVs; n=7 mice for 10A/miR-204 sEVs). **(c)** The qRT-PCR analysis of mRNA abundance of *Vhl* and *Hif1α* in fractionated primary adipocytes from each group mice (n=3 biologically independent samples per group). **(d)** Western blots showing indicated proteins in fractionated primary adipocytes from each group mice. **(e)** Body weight (n=7 mice per group) and **(f)** relative eWAT, iWAT, BAT (n=8 mice per group), gastrocnemius (GA) and tibial anterior (TA) skeletal muscle weight (n=5 mice per group) (normalized to body weight) of mice in indicated groups. **(g)** Oxygen consumption (VO_2) in sEV injected mice. Dot plots represent the 48-hour average values (n=6 mice per group). **(h)** eWAT (yellow) and iWAT (green) distribution in sEV injected NSG mice, as visualized by micro-CT (n=3 mice per group). **(i)** The qRT-PCR analysis of miR-204 abundance of adipose tissue from each group mice (n=4 mice per group). **(j)**

Sanger sequencing showing genetic KO of *mmu*-miR-204 gene in 4T1/miR-204 KO cells. Dashes indicated the deleted region. **(k)** Western blots showing indicated proteins levels in iWAT and eWAT from mice in indicated groups (n=6 mice per group for 4T1/Ctrl; n=7 mice for 4T1/miR-204 KO). **(l-m)** Body weight (n=7 mice per group), iWAT, eWAT, BAT (n=8 mice per group), GA and TA skeletal muscle (n=5 mice per group, normalized to body weight) in each group of mice. **(n)** Oxygen consumption (VO_2) in each group mice. Dot plots represent the 48-hour average values (n=6 mice per group). **(o)** Daily food intake of mice in indicated groups (n=6 mice per group). Data are presented as mean ± s.e.m; **P*<0.05, ** *P*<0.01, ****P*<0.001, *****P*<0.0001, ns: not significant. One-way ANOVA followed by Dunnett multiple comparison test was used for a, c, i. Unpaired two-tailed t-test was used for f, g, m, n and o. Two-way ANOVA followed by Bonferroni's multiple comparisons test was used for e, l. Source data and exact *P* value are provided as a Source data file.

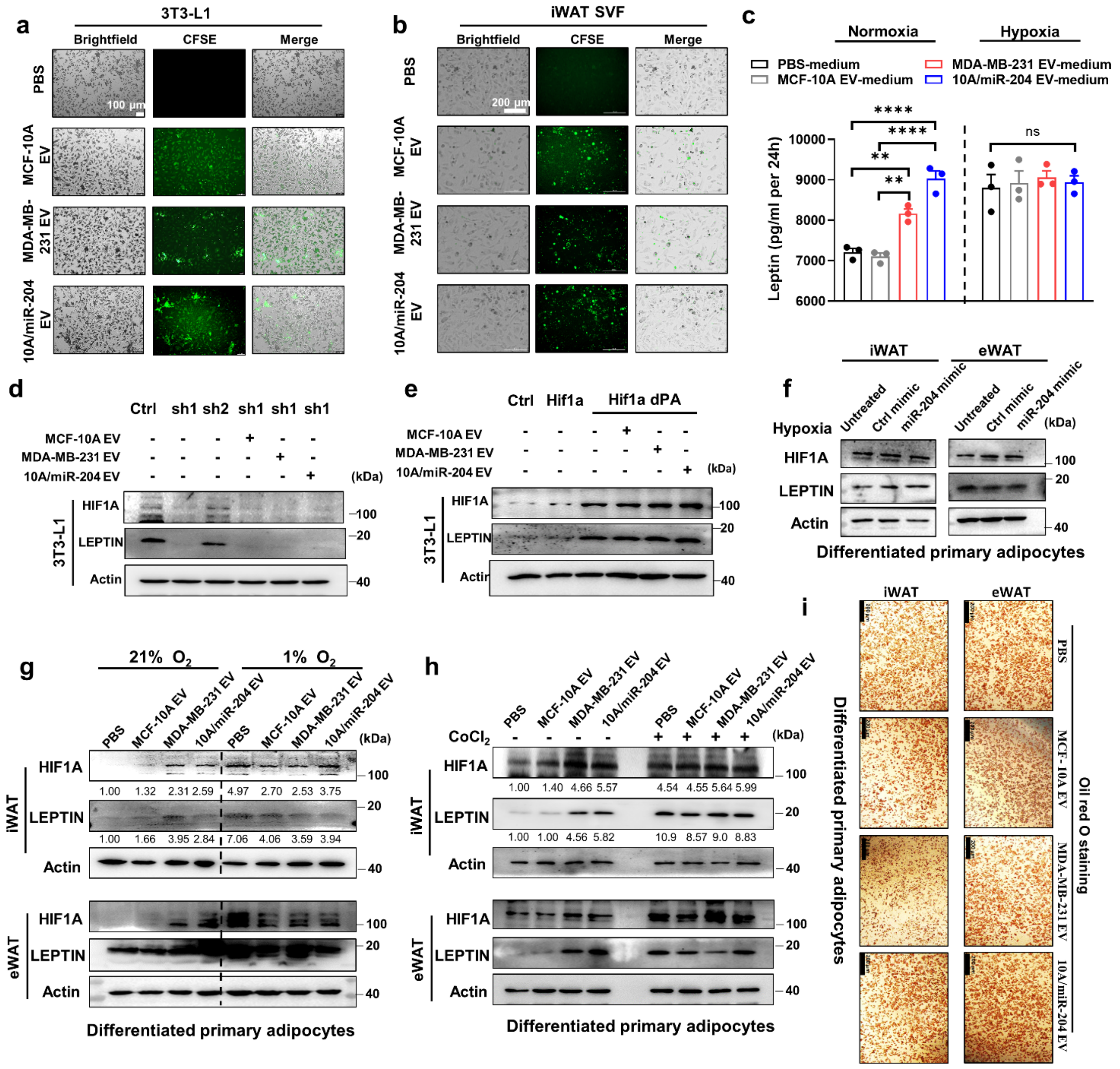

Supplementary Figure 3. Cancer-secreted miR-204 induces leptin signaling pathway in white adipose tissue.

(a-b) MCF-10A sEVs, MDA-MB-231 sEVs and 10A/miR-204 sEVs labelled CFSE (Green) treat with 3T3L-1 adipocytes and iWAT stromal vascular fraction (SVF). **(c)** The levels of leptin from cultured medium for differentiated primary adipocytes of iWAT under normoxia or hypoxia were determined by ELISA kit (n= 3 biologically independent samples per group; one-way ANOVA followed by Dunnett multiple comparison test). **(d)** Protein levels in mature 3T3-L1 treated as indicated and transfected with Ctrl shRNA or shRNA targeting HIF1A under 1% O_2. Repeated three times independently with similar results obtained. **(e)** Western blots showing indicated proteins in mature 3T3-L1 transfected with WT HIF1A or the dPA mutant with or without indicated sEVs treatment. Repeated three times independently with similar results obtained. **(f)** Western blots showing levels of indicated proteins in differentiated primary adipocytes transfected with miR-204 or control mimic under 1% O_2. Repeated three times independently with similar results obtained. **(g)** Protein levels in differentiated primary adipocytes from iWAT and eWAT treated as indicated under 21% or 1% O_2. Repeated three times independently with similar results obtained. **(h)** Western blots showing indicated proteins in differentiated primary adipocytes from iWAT and eWAT treated as indicated with or without 100 mM $CoCl_2$.

Repeated three times independently with similar results obtained. **(i)** Oil Red O staining of differentiated primary adipocytes from iWAT and eWAT treated by indicated sEVs. Repeated three times independently with similar results obtained. Scale bar, 200 μm. Data are presented as mean ± s.e.m; **P*<0.05, ** *P*<0.01, *****P*<0.0001, ns: not significant. Source data and exact *P* value are provided as a Source data file.

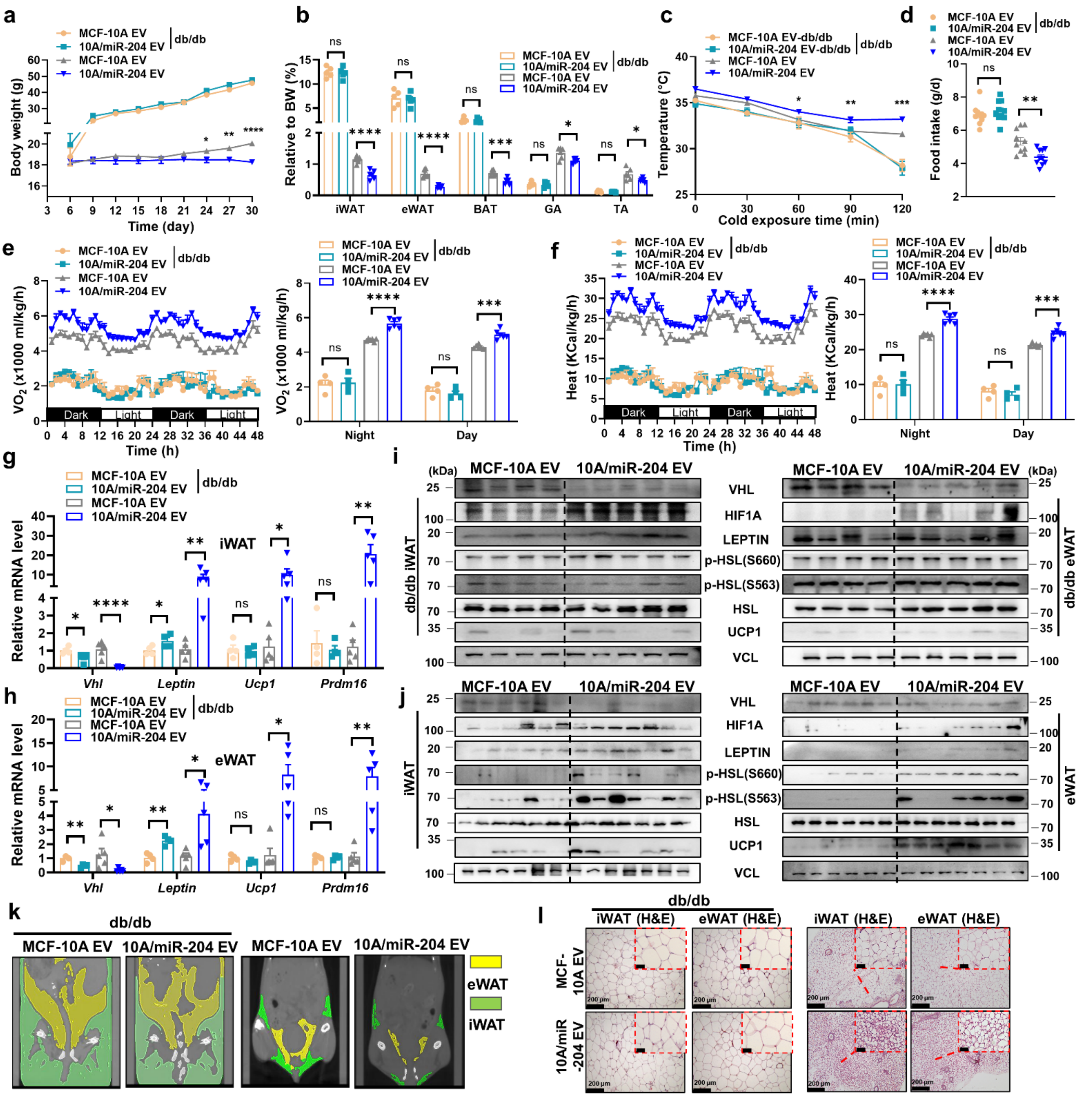

Supplementary Figure 4. Leptin receptor deficiency blocks miR-204 mediated white adipose tissue browning.

BKS-Lepr^{em2Cd479}/Gpt (BKS-db), C57BL/6J mice were tail vein injected with ~10 μg sEVs per injection per mouse (MCF-10A sEVs, 10A/miR-204 sEVs) twice a week. **(a)** Body weight (n=5 mice for db/db mice; n=6 mice for C57BL/6J mice). **(b)** Relative fat tissues weight (normalized to body weight) (n=5 mice for db/db mice; n=6 mice for C57BL/6J mice). **(c)** Rectal temperatures of the mice at different times after cold exposure (n=3 mice for db/db mice; n=6 mice for C57BL/6J mice). **(d)** Daily food intake (n=5 mice examined by 10 independent experiments for db/db mice; n=6 mice examined by 10 independent experiments for C57BL/6J mice). **(e)** Oxygen consumption (VO_2) and **(f)** heat production. Dot plots were monitored over a 48-h period in each group mice. n=4 mice for db/db mice; n=6 mice for C57BL/6J mice. **(g-h)** Relative mRNA abundance of the indicated mRNA levels in iWAT and eWAT from each group mice (n=4 mice for db/db mice; n=5 mice for C57BL/6J mice). **(i)** Western blots showing indicated proteins in iWAT and eWAT from indicated groups

of db/db mice (MCF-10A sEVs group, n=4 mice per group; 10A/miR-204 sEVs group, n=5 mice per group). **(j)** Western blots showing indicated proteins in iWAT and eWAT from indicated groups of C57BL/6J mice (MCF-10A sEVs group, n=6 mice per group; 10A/miR-204 sEVs group, n=7 mice per group). **(k)** eWAT (yellow) and iWAT (green) distribution in db/db mice and C57BL/6J mice receiving MCF-10A sEVs or 10A/miR-204 sEVs, as visualized by micro-CT (n=3 mice per group). **(l)** Representative H&E staining with iWAT/eWAT from each group mice. Repeated three times independently with similar results obtained. Scale bar, 200 μm. Data are presented as mean ± s.e.m; **P*<0.05, ***P*<0.01, ****P*<0.001, *****P*<0.0001, ns: not significant. Unpaired two-tailed t-test was used for a, b, c, d, e, f, g, h. Source data and exact *P* value are provided as a Source data file.

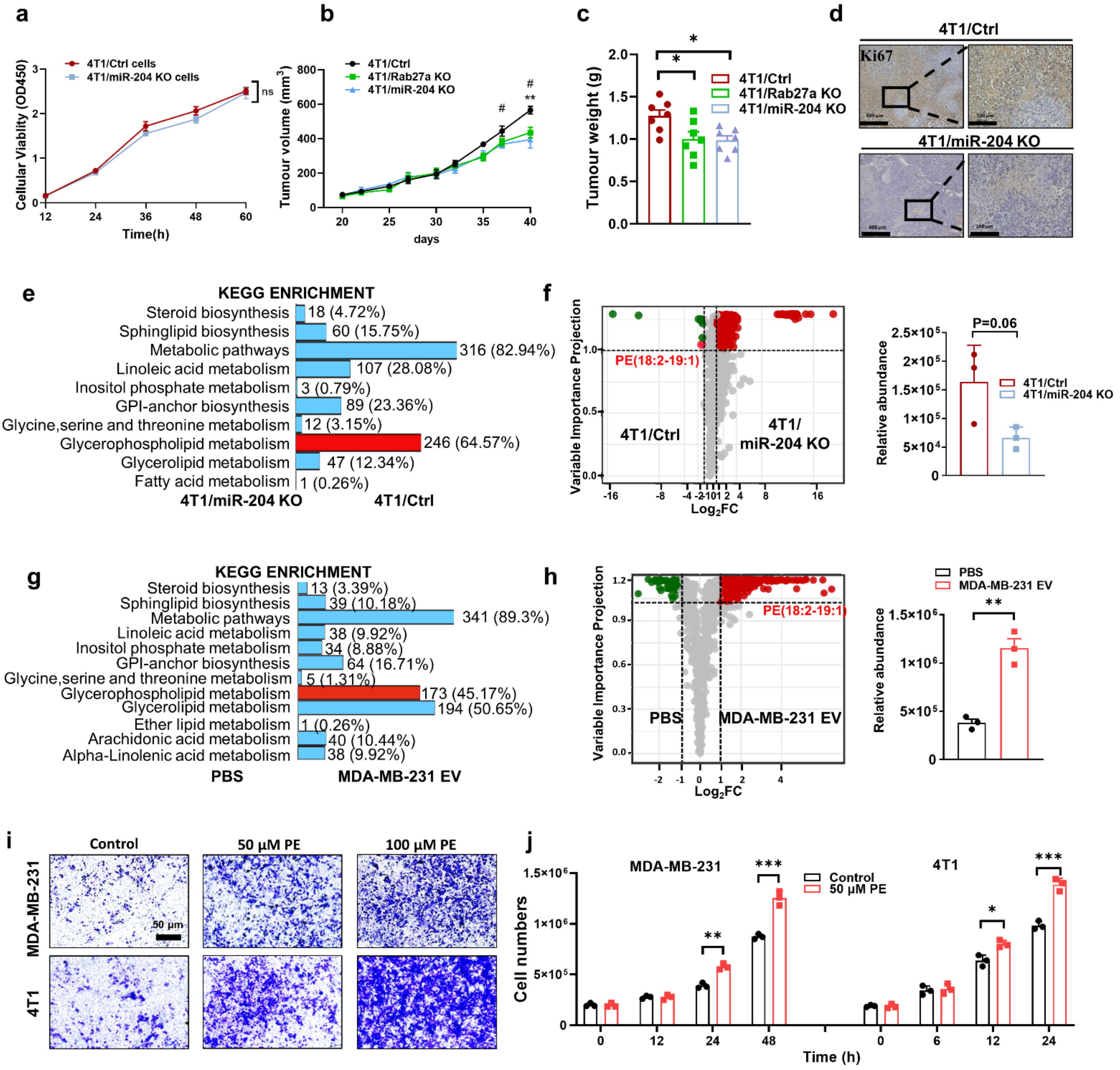

Supplementary Figure 5. Circulating miR-204 enhances phosphatidylethanolamines production to promote cancer-associated cachexia.

(a) CCK-8 was used to determine cell viability for 4T1/Ctrl and 4T1/miR-204 KO cells (n=6 biologically independent samples per group). **(b)** Tumour volumes were measured every 3 days. n=8 mice per group. **(c)** Tumour weight. n=8 mice per group. **(d)** Immunohistochemistry staining of Ki67 in 4T1/Ctrl and 4T1/miR-204 KO tumour. Repeated three times independently with similar results obtained. **(e)** KEGG pathway enrichment of metabolomics showing Glycerophospholipid metabolism and and **(f)** volcano plot deplicting levels of phosphatidylethanolamines in serum from 4T1 tumour-bearing mice and 4T1/miR-204 KO mice. n=3 mice per group. **(g)** KEGG pathway enrichment of metabolomics showing Glycerophospholipid metabolism and and **(h)** volcano plot deplicting levels of phosphatidylethanolamines in 3T3-L1 treated by MDA-MB-231 sEVs (n= 3 biologically independent samples per group). **(i)** Transwell assays for evaluating the migration and invasion capacity of MDA-MB-231 and 4T1 cells cocultured with PE. **(j)** Cell number counting under different PE concentrations (n=3 biologically independent plates). Data are presented as mean ± s.e.m; *P<0.05, **P<0.01, ***P<0.001, $^{\#}P$<0.05, ns: not significant. Source data and exact P value are provided as

a Source data file. Unpaired two-tailed t-test was used for f, h, j. Two-way ANOVA followed by Bonferroni's multiple comparisons test was used for a. One-way ANOVA was used for b and c.

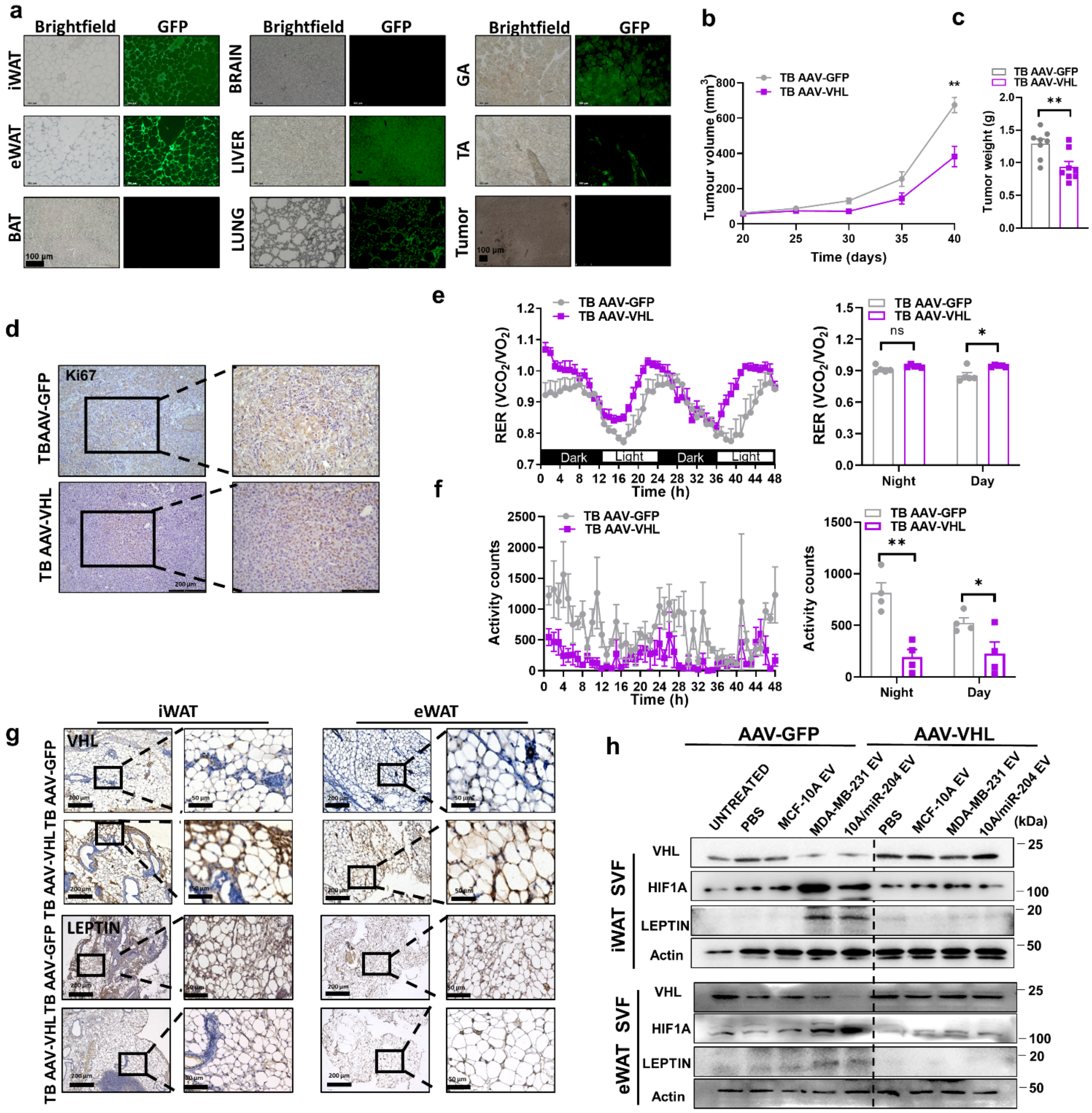

Supplementary Figure 6. Exogenous VHL expression suppresses white adipose tissue browning. **(a)** The levels of GFP (green) in tissues of tumour-bearing mice injected with AAV virus, included iWAT, eWAT, BAT, brain, liver, lung, GA, TA and tumour. Repeated three times independently with similar results obtained. Scale bar, 100 µm. **(b)** Tumour volumes were measured every 5 days (n=8 mice per group) and **(c)** tumour weight (n=8 mice per group). **(d)** Immunohistochemistry staining of Ki67 in AAV-GFP and AAV-VHL tumour. Scale bar, 200 µm. **(e-f)** Respiratory exchange ratio (RER) and physical activity were monitored over a 48-h period in the two group mice (n= 5 mice per group in e and 4 mice per group in f). **(g)** Representative immunohistochemistry staining of VHL and LEPTIN in iWAT and eWAT. Scale bar, 200 µm. **(h)** Western blots showing indicated proteins in control or transfected with AAV-Vhl in SVF cells from iWAT and eWAT. Repeated three times independently with similar results obtained. Data are presented as mean ± s.e.m; **P*<0.05, ***P*<0.01, ns: not significant. Unpaired two-tailed t-test was used for b, c, e, f. Source data and exact *P* value are provided as a Source data file.

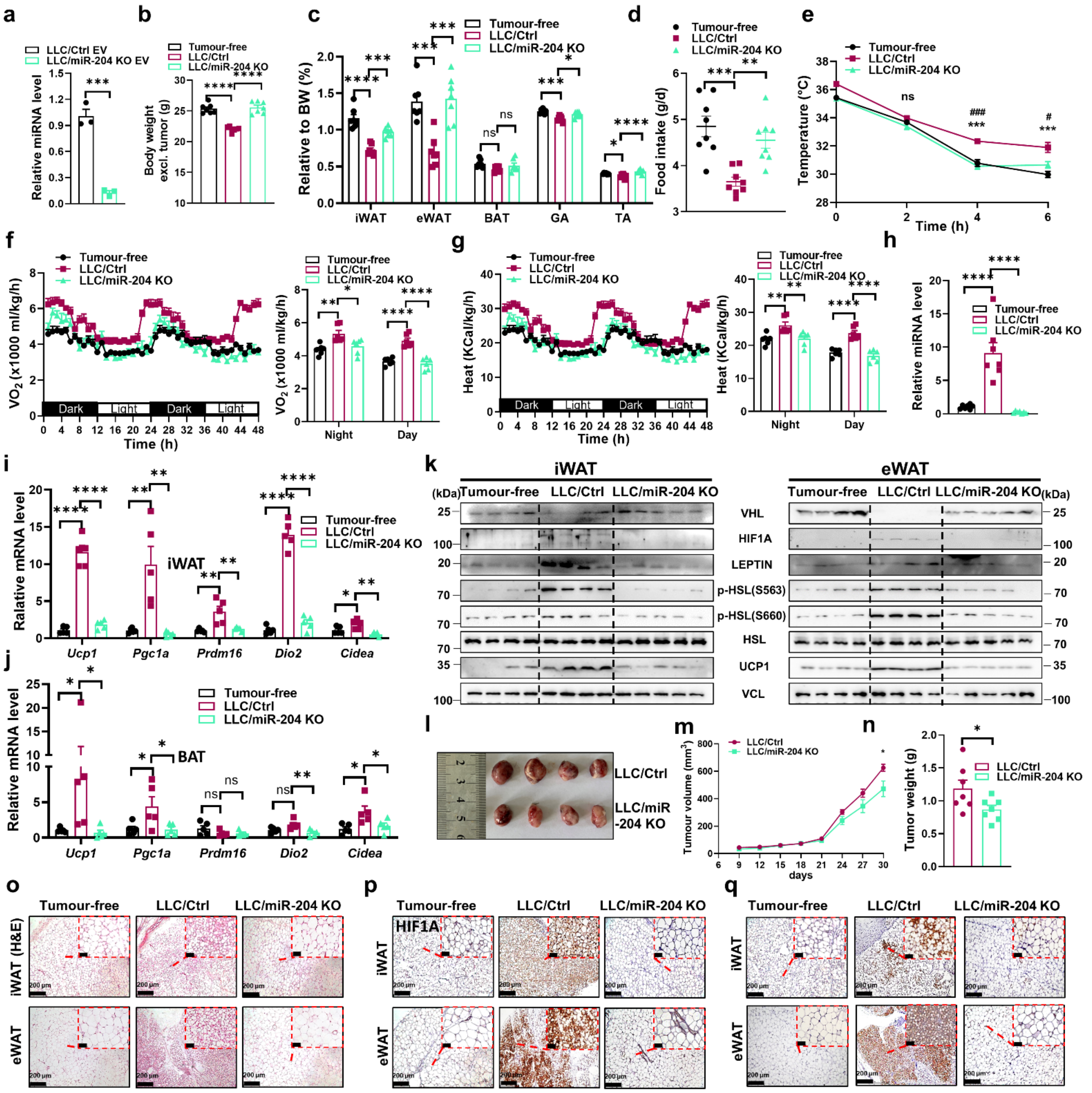

Supplementary Figure 7. Exosomal miR-204 promotes thermogenesis and lipolysis in LLC-xenografted tumour mice.

(a) The expression level of miR-204 in LLC and LLC/miR-204 KO derived sEVs. n=3 biologically independent samples per group. **(b)** Body weight (calculated by subtracting tumour weight from the total weight) of mice bearing LLC tumours (n=7 mice per group). **(c)** Relative tissues weight (normalized to body weight) (n=7 mice per group). **(d)** Daily food intake (n=8 mice per group). **(e)** Rectal temperatures of the mice at different times after cold exposure (n=5 mice per group). **(f)** Oxygen consumption (VO_2) and **(g)** heat production. Dot plots represent the 48-hour average values (n=6 mice per group). **(h)** The qRT-PCR analysis of miR-204 abundance of serum from each group mice (n=7 mice per group). **(i-j)** Relative mRNA abundance of the indicated thermogenic markers in iWAT and BAT from each group mice (n=5 mice per group). **(k)** Western blots showing indicated proteins in iWAT and eWAT from each group mice (tumour-free group, n=4 mice per group; LLC/Ctrl group, n=4 mice per group; LLC/miR-204 KO group, n=5 mice per group). **(l)** Image of excised

tumours (n=4 mice per group). **(m)** Tumour volume were measured every 3 days (n=7 mice per group) and **(n)** tumour weight (n=7 mice per group). **(o)** Representative H&E staining with iWAT/eWAT from each group mice. Repeated three times independently with similar results obtained. Scale bar, 200 μm. **(p)** Representative immunohistochemistry staining of HIF1A in iWAT and eWAT. Repeated three times independently with similar results obtained. Scale bar, 200 μm. **(q)** Representative immunohistochemistry staining of UCP1 in iWAT and eWAT. Repeated three times independently with similar results obtained. Scale bar, 200 μm. Data are presented as mean ± s.e.m; **P*<0.05, ***P*<0.01, ****P*<0.001, *****P*<0.0001, $^{\#}P<0.05$, $^{\#\#\#}P<0.001$, ns: not significant. One-way ANOVA followed by Dunnett multiple comparison test was used for b, c, d, e, f, g, h, I and j. Unpaired two-tailed t-test was used for a, m and n. Source data and exact *P* value are provided as a Source data file.

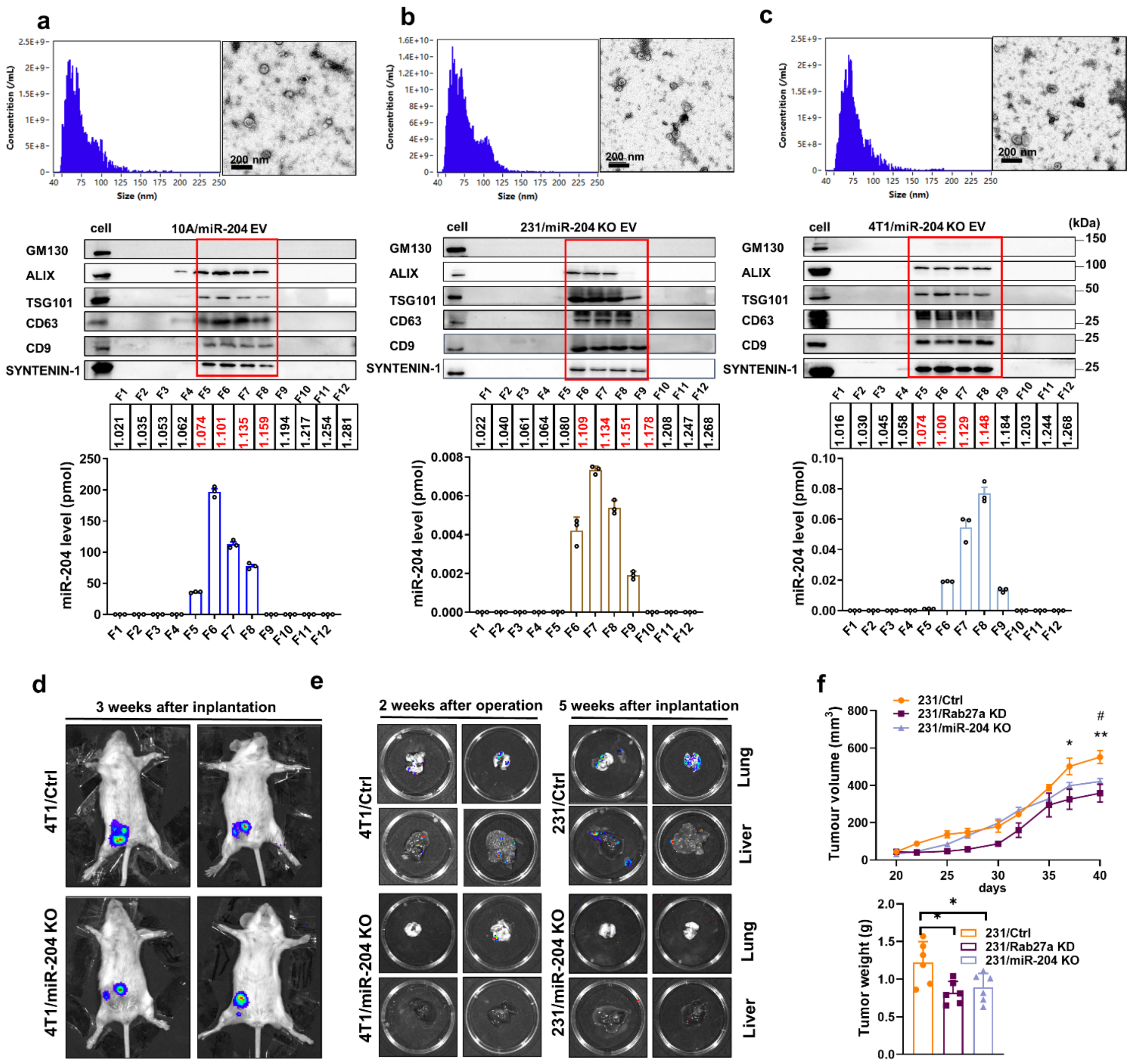

Supplementary Figure 8. sEV characterization and the effect of miR-204 sEVs on the tumour metastatic.

Characterization of sEVs derived from 10A/miR-204 **(a)**, 231/miR-204 KO **(b)** and 4T1/miR-204 KO **(c)** cells cultured medium: size distribution (top panel), western blotting (middle panel) of indicated whole cell lysates and sEVs fractions showing both positive markers and sEV negative marker (GM130) and relative miR-204 levels (bottom panel) in optiprep gradient fractions (n=3 biological replicates). In the western blot analyses, each well was loaded with 20 μg protein. **(d-e)** Bioluminescence imaging of 4T1 tumour mice at the 3 weeks after implantation, bioluminescence imaging (green) of 231 tumour mice dissected liver, lung tissues at the end of the experiment. One-way ANOVA followed by Dunnett multiple comparison test. **(f)** Tumour volume were measured every 3 days (n=6 mice per group) and tumour weight (n=6 mice per group). Data are presented as mean ± s.e.m. *P<0.05, **P<0.01, $^{\#}P$<0.05. Source data and exact P value are provided as a Source data file.

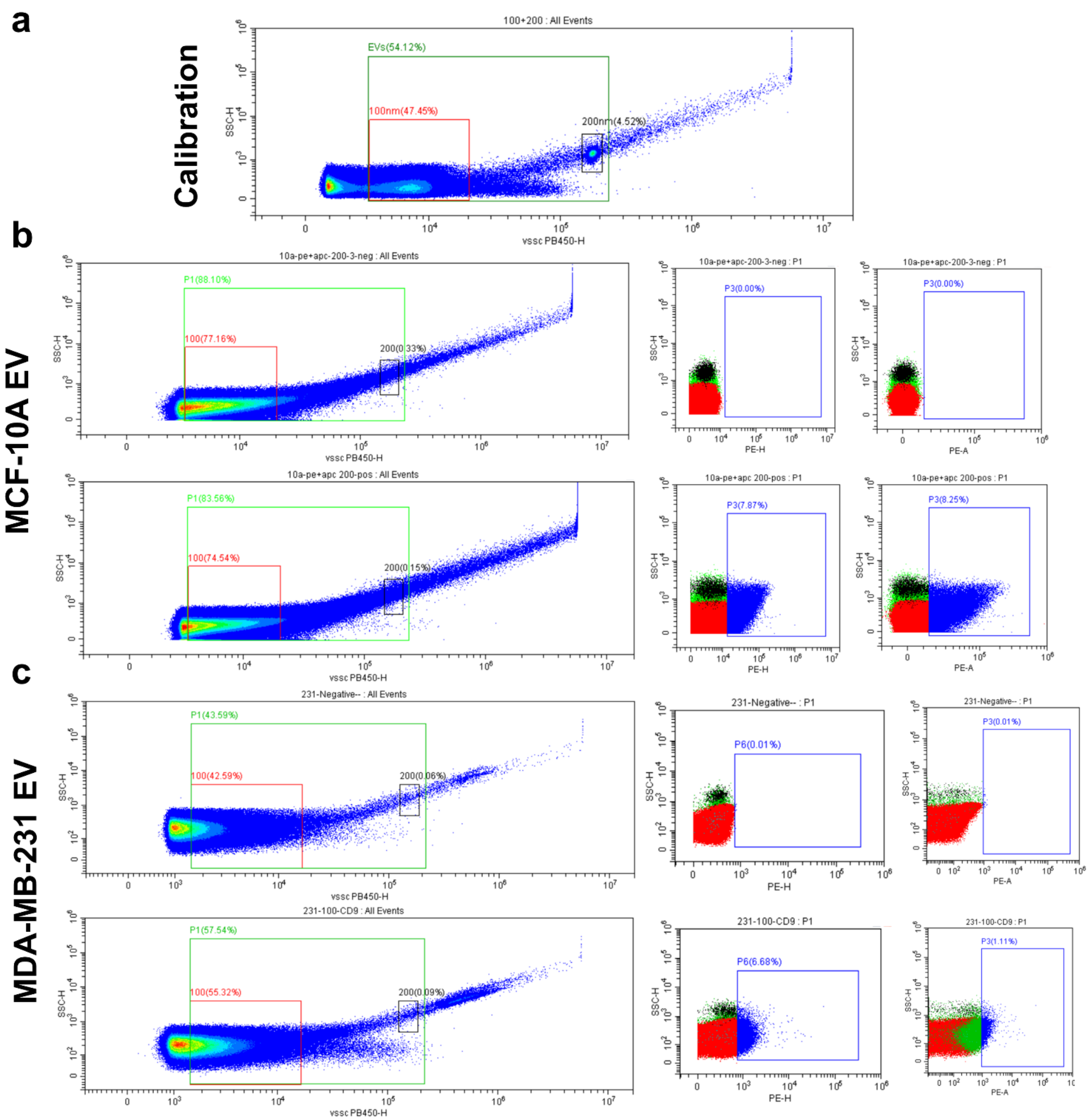

Supplementary Figure 9. Nanoflow cytometry gating strategy for MDA-MB-231 sEVs and MCF-10A sEVs.

Gating strategies used to analyze CD9 expression on the MDA-MB-231 sEV and MCF-10A sEV membranes. **(a)** Latex Beads (100 nm and 200 nm mean particle size) (PCS Control Mixed Kit, Beckman Coulter) are characterized and serve as a vesicle size standard. **(b-c)** Representative sEV samples immunostained with the indicated PE-labelled CD9 antibodies.

Supplementary Table 1: Predicted miRNA regulators of VHL.

miRNA	Position in the UTR	seed match	context++ score	context++ score percentile	weighted context++ score	conserved branch length	Pct
hsa-miR-223-3p	146-152	7mer-m8	-0.25	91	-0.25	3.383	0.35
hsa-miR-211-5p	631-638	8mer	-0.37	98	-0.37	3.269	0.32
***hsa*-miR-204-5p**	**631-638**	**8mer**	**-0.37**	**98**	**-0.37**	**3.269**	**0.32**
hsa-miR-5590-3p	1657-1664	8mer	-0.16	96	-0.16	2.22	< 0.1
hsa-miR-142-5p	1657-1664	8mer	-0.17	96	-0.16	2.22	< 0.1
hsa-miR-340-5p	1658-1665	8mer	-0.08	96	-0.08	2.381	N/A
hsa-miR-21-5p	1689-1695	7mer-m8	-0.2	91	-0.19	3.157	0.2
hsa-miR-590-5p	1689-1695	7mer-m8	-0.15	91	-0.14	3.157	0.2
hsa-miR-101-3p.1	3506-3512	7mer-m8	-0.26	95	-0.25	2.964	0.52
hsa-miR-181b-5p	3585-3592	8mer	-0.24	95	-0.19	3.333	0.22
hsa-miR-181d-5p	3585-3592	8mer	-0.24	95	-0.19	3.333	0.22
hsa-miR-181c-5p	3585-3592	8mer	-0.25	95	-0.19	3.333	0.22
hsa-miR-181a-5p	3585-3592	8mer	-0.24	95	-0.19	3.333	0.22
hsa-miR-4262	3585-3592	8mer	-0.13	91	-0.1	3.333	0.22
hsa-miR-374c-5p	3589-3596	8mer	-0.17	89	-0.13	3.625	N/A
hsa-miR-655-3p	3589-3596	8mer	-0.17	88	-0.13	3.625	N/A
hsa-miR-29a-3p	3684-3690	7mer-m8	-0.43	97	-0.29	4.977	0.86
hsa-miR-29c-3p	3684-3690	7mer-m8	-0.43	97	-0.29	4.977	0.86
hsa-miR-29b-3p	3684-3690	7mer-m8	-0.43	97	-0.29	4.977	0.86
hsa-miR-329-3p	3710-3716	7mer-m8	-0.23	98	0	2.993	N/A
hsa-miR-362-3p	3710-3716	7mer-m8	-0.22	97	0	2.993	N/A

Supplementary Table 2: Oligonucleotide primers for qRT-PCR analysis.

Species	Gene	Forward primer (5'-3')	Reverse primer (5'-3')
Mouse	*Leptin*	GAGACCCCTGTGTCGGTTC	CTGCGTGTGTGAAATGTCATTG
	Npy	TACTCCGCTCTGCGACACTACA	GGCGTTTTCTGTGCTTTCCTTCA
	Agrp	GCCTCAAGAAGACAACTGCAGAC	AAGCAGGACTCGTGCAGCCTTA
	Pgc1α	GAATCAAGCCACTACAGACACCG	CATCCCTCTTGAGCCTTTCGTG
	Dio2	GGTGGTCAACTTTGGTTCAGCC	AAGTCAGCCACCGAGGAGAACT
	Prdm16	ATCCACAGCACGGTGAAGCCAT	ACATCTGCCCACAGTCCTTGCA
	Ucp1	AGGCTTCCAGTACCATTAGGT	CTGAGTGAGGCAAAGCTGATTT
	Mt-Co1	CACCAGTCAATCCCTGTTGTTACT	GGTAGTTGTCGAGGCCAAAGC
	Ndufv1	GTGGAGGAAGAGATGTCTGTGC	ATGAGCCACCAGGAATCACAGC
	Vhl	GTTTGTGCCATCCCTCAATGTCG	ACCTGACGATGTCCAGTCTCCT
	Hif1a	CCTGCACTGAATCAAGAGGTTGC	CCATCAGAAGGACTTGCTGGCT
	Cidea	GGTGGACACAGAGGAGTTCTTTC	CGAAGGTGACTCTGGCTATTCC
	Actin	CATTGCTGACAGGATGCAGAAGG	TGCTGGAAGGTGGACAGTGAGG
HUMAN	*VHL*	GCTGTGCCCATCCAAAAAGTCC	CCCAGGAATGAAGTCCAAACCG
	LEPTIN	GACACACGATGGGCTTCTGGTT	ACAACCTGGAGGCATCGCTCTT
	ACTIN	CACCATTGGCAATGAGCGGTTC	AGGTCTTTGCGGATGTCCACGT

Supplementary Table 3: Antibodies used in this study.

Antibodies	SOURCE	IDENTIFIER	Dilutions/amounts	Manufacturer's website including validation information
Rab27a (E-8) Mouse monoclonal antibodies	Santa Cruz	Cat# sc-74586	1:100 (WB)	https://www.scbt.com/zh/p/rab-27a-antibody-e-8?requestFrom=search
Alix Rabbit polyclonal antibodies	Proteintech	Cat# 12422-1-AP	1:5000 (WB)	https://www.ptgcn.com/products/PDCD6IP-Antibody-12422-1-AP.htm
CD9 Rabbit polyclonal antibodies	Proteintech	Cat# 20597-1-AP	1:1000 (WB)	https://www.ptgcn.com/products/CD9-Antibody-20597-1-AP.htm
CD63 Rabbit polyclonal antibody	Proteintech	Cat# 25682-1-AP	1:200 (WB)	https://www.ptgcn.com/products/CD63-Antibody-25682-1-AP.htm
CD63 (MX-49.129.5) mouse monoclonal antibodies	Santa Cruz	Cat# sc-5275	1:100 (WB)	https://www.scbt.com/zh/p/cd63-antibody-mx-49-129-5?requestFrom=search
TSG101 Rabbit polyclonal antibody	Proteintech	Cat# 28283-1-AP	1:2000 (WB)	https://www.ptgcn.com/products/TSG101-Antibody-28283-1-AP.htm
GOLGA2/GM130 Rabbit polyclonal	Proteintech	Cat# 11308-1-AP	1:2000 (WB)	https://www.ptgcn.com/products/GOLGA2,GM130-Antibody-11308-1-AP.htm
Syntenin-1 Rabbit polyclonal antibody	Proteintech	Cat# 22399-1-AP	1:500 (WB)	https://www.ptgcn.com/products/SDCBP-Antibody-22399-1-AP.htm
ACTIN (2D4H5) Mouse monoclonal antibodies	Proteintech	Cat# 66009-1-Ig	1:20000 (WB)	https://www.ptgcn.com/products/Pan-Actin-Antibody-66009-1-Ig.htm
HIF1a Rabbit polyclonal antibody	Proteintech	Cat# 20960-1-AP	1:2000 (WB); 1:200 (IHC)	https://www.ptgcn.com/products/HIF1A-Antibody-20960-1-AP.htm
HIF1 alpha (AFfirm8002(AFB 17813)) Mouse monoclonal Antibody	Affinity	Cat# BF8002	1:1000 (WB)	https://www.affbiotech.cn/goods-16579-BF8002-HIF1_alpha_Mouse_monoclonal_Antibody.html
Anti-UCP1	Abcam	Cat# ab10983	1:1000 (WB); 1:500 (IHC)	https://www.abcam.cn/ucp1-antibody-ab10983.html
Vinculin Rabbit mAb	Abclonal	Cat# A23468	1:1000 (WB)	https://abclonal.com.cn/catalog/A23468
CREB (48H2) Rabbit mAb	Cell Signaling Technology	Cat# 9197	1:1000 (WB)	https://www.cellsignal.cn/products/primary-antibodies/creb-48h2-rabbit-mab/9197?site-search-type=Products&N=4294956287&Ntt=9197&fromPage=plp&_requestid=3098024
Phospho-CREB (Ser133) (87G3) Rabbit mAb	Cell Signaling Technology	Cat# 9198	1:1000 (WB)	https://www.cellsignal.cn/products/primary-antibodies/phospho-creb-ser133-87g3-rabbit-mab/9198?site-search-type=Products&N=4294956287&Ntt=9198&fromPage=plp&_requestid=3100133
ATGL Antibody	Cell Signaling Technology	Cat# 2138	1:1000 (WB)	https://www.cellsignal.cn/products/primary-antibodies/atgl-antibody/2138?site-search-type=Products&N=4294956287&Ntt=2138&fromPage=plp&_requestid=3100495
HSL Antibody	Cell Signaling Technology	Cat# 4107	1:1000 (WB)	https://www.cellsignal.cn/products/primary-antibodies/hsl-antibody/4107?site-search-type=Products&N=4294956287&Ntt=4107&fromPage=plp&_requestid=3100652
Phospho-HSL (Ser563) Antibody	Cell Signaling Technology	Cat# 4139	1:1000 (WB)	https://www.cellsignal.cn/products/primary-antibodies/phospho-hsl-ser563-antibody/4139?site-search-type=Products&N=4294956287&Ntt=4139&fromPage=plp&_requestid=3102740
Phospho-HSL (Ser660) Antibody	Cell Signaling Technology	Cat# 45804	1:1000 (WB)	https://www.cellsignal.cn/products/primary-antibodies/phospho-hsl-ser660-antibody/45804?site-search-type=Products&N=4294956287&Ntt=45804&fromPage=plp&_requestid=3103130
VHL Antibody	Affinity	Cat# AF6292	1:1000 (WB)	https://www.affbiotech.cn/goods-1890-AF6292-VHL_Antibody.html
Leptin Antibody	Affinity	Cat# DF8583	1:1000 (WB);1:100 (IHC)	https://www.affbiotech.cn/goods-12056-DF8583-Leptin_Antibody.html
STAT3 Antibody	Affinity	Cat# AF6294	1:1000 (WB)	https://www.affbiotech.cn/goods-1892-AF6294-STAT3_Antibody.html
Phospho-STAT3 (Tyr705) Antibody	Affinity	Cat# AF3293	1:1000 (WB)	https://www.affbiotech.cn/goods-1458-AF3293-Phospho_STAT3_Tyr705_Antibody.html

Ki67 Antibody	Affinity	Cat# AF0198	1:100 (IHC)	https://www.affbiotech.cn/goods-897-AF0198-Ki67_Antibody.html
Sheep Anti-Digoxigenin Fab fragments Antibody, AP Conjugated	Roche	Cat# 11093274910	5mU/ml (ISH)	https://www.sigmaaldrich.cn/CN/zh/product/roche/11093274910
Goat anti-Mouse IgG (H+L) Secondary Antibody	Thermo Fisher Scientific	Cat# 31430	1:5000 (WB)	https://www.thermofisher.cn/cn/zh/antibody/product/Goat-anti-Mouse-IgG-H-L-Secondary-Antibody-Polyclonal/31430
Goat anti-Rabbit IgG (H+L) Secondary Antibody	Thermo Fisher Scientific	Cat# 31460	1:10000 (WB)	https://www.thermofisher.cn/cn/zh/antibody/product/Goat-anti-Rabbit-IgG-H-L-Secondary-Antibody-Polyclonal/31460
PE anti-human CD9 Antibody	BioLegend	Cat# 312106	1:50 (FC)	https://www.biolegend.com/en-us/products/pe-anti-human-cd9-antibody-2213

Supplementary Table 4: Clinical information included in this study.

Patient No.	Medical record No.	Age	Height(cm)	Weight(kg)	BMI	Grade	ER	PR	HER2	Notes
1	10228646	71	160	50	19.53125	Moderately	+	+	+	
2	10223146	80	158	59	23.63403	Moderately	+	+	+	
3	10222574	69	158	52	20.83	Poorly	+	+	++	
4	10220569	71	157	55	22.31328	Moderately	+	+	+	
5	10221730	61	155	56	23.30905	Moderately	-	-	+++	
6	10221708	63	148	48	21.91381	Poorly	-	-	-	
7	10213169	51	156	51	20.95661	Poorly	+	+	+	
8	10210360	53	158	55	22.03173	Moderately	+	+	-	
9	10208290	47	158	54	21.63115	Poorly	+	-	+	
10	10196171	52	158	56	22.4323	Poorly	-	-	++	
11	10170776	74	170	62	21.45329	Moderately	+	+	-	
12	10189802	53	170	71	24.56747	Moderately	+	+	++	
13	10174233	70	156	55	22.60026	Poorly	-	-	-	
14	10173299	42	162	62	23.62445	Poorly	-	-	-	
15	10191000	38	153	42.5	18.15541	Poorly	-	-	-	cachexia
16	10197923	40	155	41	17.06556	Poorly	-	-	-	cachexia
17	10195792	56	158	52	20.83	Poorly	-	-	-	
18	10194890	58	164	59	21.93635	Moderately	+	+	+	
19	10190185	58	168	55	19.48696	Moderately	-	-	+	
20	10251715	52	168	67	23.73866	Poorly	-	-	-	
21	10256435	68	160	58	22.65625	Poorly	-	-	+	
22	10235511	31	167	44	15.77683	Poorly	-	-	-	cachexia
23	10254206	66	160	45	17.57813	Poorly	-	-	-	cachexia
24	10254249	33	162	162	17.90886	Poorly	-	-	-	cachexia
25	10232071	30	160	47	18.35938	Poorly	-	-	-	cachexia
26	10248783	50	159	47	18.59104	Poorly	-	-	+	
27	10232278	32	160	48	18.75					
28	10253886	70	158	78	31.24499	Moderately	+	+	+	
29	10241992	35	163	82.5	31.05123					
30	10228564	67	165	80	29.38476	Moderately	+	+	+	
31	10190333	58	155	68	28.30385					
32	10190975	70	155	68	28.30385	Moderately	+	+	+	
33	10232250	58	158	70	28.04038					
34	10276291	44	158	70	28.04038	Moderately	+	+	++	
35	10274128	70	164	75	27.88519					
36	10210360	53	158	55	22.03173	Moderately	+	+	-	
37	10213169	51	156	51	20.95661	Moderately	+	+	+	
38	10214357	64	155	60	24.97399	Moderately	+	+	-	
39	10216929	36	165	65	23.87511	Moderately	+	+	++	
40	10221062	73	164	60	22.30815	Moderately	+	+	+	
41	10221708	63	148	48	21.91381	Poorly	-	-	-	
42	10180033	37	162	69	26.29172	Moderately	+	+	-	
43	10178763	52	150	55.5	24.66667	Moderately	+	+	+	
44	10180782	65	162	61.5	23.43393	Moderately	+	+	+	
45	10181069	52	164	63	23.42356	Moderately	+	+	-	
46	10181305	38	170	65	22.49135	Moderately	+	+	+++	
