# Supplementary material for: Cancer-cell-secreted miR-204-5p induces leptin signalling pathway in white adipose tissue to promote cancer-associated cachexia
Source: Nat Commun. 2023 Aug 24;14:5179. doi: 10.1038/s41467-023-40571-9 (PMC10449837; doi:10.1038/s41467-023-40571-9)
Supplement: Supplementary file 3 — Reporting Summary [file 41467_2023_40571_MOESM3_ESM.pdf]

nature portfolio

Corresponding author(s): Wei Yan/Yancheng Xu

Last updated by author(s): Jul 17, 2023

Reporting Summary

Nature Portfolio wishes to improve the reproducibility of the work that we publish. This form provides structure for consistency and transparency in reporting. For further information on Nature Portfolio policies, see our Editorial Policies and the Editorial Policy Checklist.

Statistics

For all statistical analyses, confirm that the following items are present in the figure legend, table legend, main text, or Methods section.

n/a	Confirmed	
☐	☒	The exact sample size (*n*) for each experimental group/condition, given as a discrete number and unit of measurement
☐	☒	A statement on whether measurements were taken from distinct samples or whether the same sample was measured repeatedly
☐	☒	The statistical test(s) used AND whether they are one- or two-sided<br>*Only common tests should be described solely by name; describe more complex techniques in the Methods section.*
☐	☒	A description of all covariates tested
☐	☒	A description of any assumptions or corrections, such as tests of normality and adjustment for multiple comparisons
☐	☒	A full description of the statistical parameters including central tendency (e.g. means) or other basic estimates (e.g. regression coefficient) AND variation (e.g. standard deviation) or associated estimates of uncertainty (e.g. confidence intervals)
☐	☒	For null hypothesis testing, the test statistic (e.g. *F*, *t*, *r*) with confidence intervals, effect sizes, degrees of freedom and *P* value noted<br>*Give P values as exact values whenever suitable.*
☒	☐	For Bayesian analysis, information on the choice of priors and Markov chain Monte Carlo settings
☒	☐	For hierarchical and complex designs, identification of the appropriate level for tests and full reporting of outcomes
☒	☐	Estimates of effect sizes (e.g. Cohen's *d*, Pearson's *r*), indicating how they were calculated

Our web collection on statistics for biologists contains articles on many of the points above.

Software and code

Policy information about availability of computer code

Data collection	Bio-Rad CFX Manager version 3.1 was used to collect real-time PCR data; Seahorse:Wave Software 2.4;flow cytometry analysis:CytExpert 2.0 and NF Profession 2.0.
Data analysis	Image J version 1.52 were used for used to quantify western blot data.<br>Data were graphed and statistically tested using GraphPad Prism 9.4.1.<br>GSEA version 4.2.0 was used for gene set enrichment analysis of RNA-seq data.<br>HIASAT (v0.6.1), Bowtie (2.4.4), fastx toolkit (0.0.13) were used in RNA-seq data analysis.<br>GMS 3 used for TEM data.<br>Aperio ImageScope (12.4.3.5008) was used for HE, IHC and ISH data.

For manuscripts utilizing custom algorithms or software that are central to the research but not yet described in published literature, software must be made available to editors and reviewers. We strongly encourage code deposition in a community repository (e.g. GitHub). See the Nature Portfolio guidelines for submitting code & software for further information.

Data

Policy information about availability of data

All manuscripts must include a data availability statement. This statement should provide the following information, where applicable:

- Accession codes, unique identifiers, or web links for publicly available datasets
- A description of any restrictions on data availability
- For clinical datasets or third party data, please ensure that the statement adheres to our policy

RNA-seq data generated in this study has been deposited in the NCBI Gene Expression Omnibus (GEO) (https://www.ncbi.nlm.nih.gov/geo/query/acc.cgi?acc=GSE222380). LC-MS/MS-detected metabolites enrichment pathways have been deposited in Figshare (10.6084/m9.figshare.23684127). The previously published GEO data set GSE 50429 was reanalysed for miRNA levels in MDA-MB-231 EV and MCF-10A EV, and other miRNA level data have been deposited in Figshare (10.6084/m9.figshare.23691432). Source data are provided with this paper.

Human research participants

Policy information about studies involving human research participants and Sex and Gender in Research.

Reporting on sex and gender	Five female specimen in total for IHC; thirty-six female subjects in total for for western blots and RT-qPCR and thirty-six female person for ELISA quantification. No gender related issues are applied to this analysis.
Population characteristics	For IHC cancer tissues were obtained from 5 female patients median age 52 (range 37-68 years). For western blots, adipose tissues were obtained from 6 female non-cancer patients median age 59 (range 32-69 years) and 6 female cancer patients median age 63 (range 36-73 years). For RT-qPCR, serum obtained from 6 female non-cancer patients median age 54 (range 41-65 years) and 18 female cancer patients median age 53 (range 30-70 years). For Elisa Kit we collected 18 female non-cancer patients median age 49 (range 30-65 years) and 18 female cancer patients median age 51 (range 38-80 years).
Recruitment	Resection material was collected from primary tumors during surgery. The presence of carcinoma was proven on histopathology.
Ethics oversight	The study was conducted in accordance with the declaration of Helsinki Declaration was approved by the Clinical Research Ethical Committee of Renmin Hospital of Wuhan University. All participants provided informed consent.

Note that full information on the approval of the study protocol must also be provided in the manuscript.

Field-specific reporting

Please select the one below that is the best fit for your research. If you are not sure, read the appropriate sections before making your selection.

☒ Life sciences ☐ Behavioural & social sciences ☐ Ecological, evolutionary & environmental sciences

For a reference copy of the document with all sections, see nature.com/documents/nr-reporting-summary-flat.pdf

Life sciences study design

All studies must disclose on these points even when the disclosure is negative.

Sample size	Sample size was generally chosen based on preliminary data indicating the variance within each group and the differences between groups.
Data exclusions	No samples/animals/data were excluded from the analysis.
Replication	All reported experimental findings were reliably reproduced at least two independent experiments or by multiple biologically independent replicates.
Randomization	All mice/samples were randomized before experiments.
Blinding	Data collection and analysis were performed blinded to group allocation.

Reporting for specific materials, systems and methods

We require information from authors about some types of materials, experimental systems and methods used in many studies. Here, indicate whether each material, system or method listed is relevant to your study. If you are not sure if a list item applies to your research, read the appropriate section before selecting a response.

Materials & experimental systems

n/a	Involved in the study
☐	☒ Antibodies
☐	☒ Eukaryotic cell lines
☒	☐ Palaeontology and archaeology
☐	☒ Animals and other organisms
☒	☐ Clinical data
☒	☐ Dual use research of concern

Methods

n/a	Involved in the study
☒	☐ ChIP-seq
☐	☒ Flow cytometry
☒	☐ MRI-based neuroimaging

Antibodies

Antibodies used

Rab27a (E-8) Mouse monoclonal antibodies Santa Cruz Cat# sc-74586 1:100 (WB) https://www.scbt.com/zh/p/rab-27a-antibody-e-8?requestFrom=search
Alix Rabbit polyclonal antibodies Proteintech Cat# 12422-1-AP 1:5000 (WB) https://www.ptgcn.com/products/PDCD6IP-Antibody-12422-1-AP.htm
CD9 Rabbit polyclonal antibodies Proteintech Cat# 20597-1-AP 1:1000 (WB) https://www.ptgcn.com/products/CD9-Antibody-20597-1-AP.htm
CD63 Rabbit polyclonal antibody Proteintech Cat# 25682-1-AP 1:200 (WB) https://www.ptgcn.com/products/CD63-Antibody-25682-1-AP.htm
CD63 (MX-49.129.5) mouse monoclonal antibodies Santa Cruz Cat# sc-5275 1:100 (WB) https://www.scbt.com/zh/p/cd63-antibody-mx-49-129-5?requestFrom=search
TSG101 Rabbit polyclonal antibody Proteintech Cat# 28283-1-AP 1:2000 (WB) https://www.ptgcn.com/products/TSG101-Antibody-28283-1-AP.htm
GOLGA2/GM130 Rabbit polyclonal Proteintech Cat# 11308-1-AP 1:2000 (WB) https://www.ptgcn.com/products/GOLGA2,GM130-Antibody-11308-1-AP.htm
Syntenin-1 Rabbit polyclonal antibody Proteintech Cat# 22399-1-AP 1:500 (WB) https://www.ptgcn.com/products/SDCBP-Antibody-22399-1-AP.htm
ACTIN (2D4H5) Mouse monoclonal antibodies Proteintech Cat# 66009-1-Ig 1:20000 (WB) https://www.ptgcn.com/products/Pan-Actin-Antibody-66009-1-Ig.htm
HIF1a Rabbit polyclonal antibody Proteintech Cat# 20960-1-AP 1:2000 (WB);1:200 (IHC) https://www.ptgcn.com/products/HIF1A-Antibody-20960-1-AP.htm
HIF1 alpha (AFfirm8002(AFB17813)) Mouse monoclonal Antibody Affinity Cat# BF8002 1:1000 (WB) https://www.affbiotech.cn/goods-16579-BF8002-HIF1_alpha_Mouse_monoclonal_Antibody.html
Anti-UCP1 Abcam Cat# ab10983 1:1000 (WB);1:500 (IHC) https://www.abcam.cn/ucp1-antibody-ab10983.html
Vinculin Rabbit mAb Abclonal Cat# A23468 1:1000 (WB) https://abclonal.com.cn/catalog/A23468
CREB (48H2) Rabbit mAb Cell Signaling Technology Cat# 9197 1:1000 (WB) https://www.cellsignal.cn/products/primary-antibodies/creb-48h2-rabbit-mab/9197?site-search-type=Products&N=4294956287&Ntt=9197&fromPage=plp&_requestid=3098024
Phospho-CREB (Ser133) (87G3) Rabbit mAb Cell Signaling Technology Cat# 9198 1:1000 (WB) https://www.cellsignal.cn/products/primary-antibodies/phospho-creb-ser133-87g3-rabbit-mab/9198?site-search-type=Products&N=4294956287&Ntt=9198&fromPage=plp&_requestid=3100133
ATGL Antibody Cell Signaling Technology Cat# 2138 1:1000 (WB) https://www.cellsignal.cn/products/primary-antibodies/atgl-antibody/2138?site-search-type=Products&N=4294956287&Ntt=2138&fromPage=plp&_requestid=3100495
HSL Antibody Cell Signaling Technology Cat# 4107 1:1000 (WB) https://www.cellsignal.cn/products/primary-antibodies/hsl-antibody/4107?site-search-type=Products&N=4294956287&Ntt=4107&fromPage=plp&_requestid=3100652
Phospho-HSL (Ser563) Antibody Cell Signaling Technology Cat# 4139 1:1000 (WB) https://www.cellsignal.cn/products/primary-antibodies/phospho-hsl-ser563-antibody/4139?site-search-type=Products&N=4294956287&Ntt=4139&fromPage=plp&_requestid=3102740
Phospho-HSL (Ser660) Antibody Cell Signaling Technology Cat# 45804 1:1000 (WB) https://www.cellsignal.cn/products/primary-antibodies/phospho-hsl-ser660-antibody/45804?site-search-type=Products&N=4294956287&Ntt=45804&fromPage=plp&_requestid=3103130
VHL Antibody Affinity Cat# AF6292 1:1000 (WB) https://www.affbiotech.cn/goods-1890-AF6292-VHL_Antibody.html
Leptin Antibody Affinity Cat# DF8583 1:1000 (WB);1:100 (IHC) https://www.affbiotech.cn/goods-12056-DF8583-Leptin_Antibody.html
STAT3 Antibody Affinity Cat# AF6294 1:1000 (WB) https://www.affbiotech.cn/goods-1892-AF6294-STAT3_Antibody.html
Phospho-STAT3 (Tyr705) Antibody Affinity Cat# AF3293 1:1000 (WB) https://www.affbiotech.cn/goods-1458-AF3293-Phospho_STAT3_Tyr705_Antibody.html
Ki67 Antibody Affinity Cat# AF0198 1:100 (IHC) https://www.affbiotech.cn/goods-897-AF0198-Ki67_Antibody.html
Sheep Anti-Digoxigenin Fab fragments Antibody, AP Conjugated Roche Cat# 11093274910 5mU/ml (ISH) https://www.sigmaaldrich.cn/CN/zh/product/roche/11093274910
Goat anti-Mouse IgG (H+L) Secondary Antibody Thermo Fisher Scientific Cat# 31430 1:5000 (WB) https://www.thermofisher.cn/cn/zh/antibody/product/Goat-anti-Mouse-IgG-H-L-Secondary-Antibody-Polyclonal/31430
Goat anti-Rabbit IgG (H+L) Secondary Antibody Thermo Fisher Scientific Cat# 31460 1:10000 (WB) https://www.thermofisher.cn/cn/zh/antibody/product/Goat-anti-Rabbit-IgG-H-L-Secondary-Antibody-Polyclonal/31460
PE (HI9a) anti-human CD9 Antibody BioLegend Cat# 312106 1:50 (FC) https://www.biolegend.com/en-us/products/pe-anti-human-cd9-antibody-2213

Validation

All antibodies used were commercially available and tested by manufacturers. Their validation statements are available on the manufacturer's website.

Rab27a(E-8) Mouse monoclonal antibodies Santa Cruz Cat# sc-74586; WB/IP/IF/IHC(P)/ELISA; mouse, rat, human
https://www.scbt.com/zh/p/rab-27a-antibody-e-8?requestFrom=search
Alix Rabbit polyclonal antibodies Proteintech Cat# 12422-1-AP; IF, IHC, IP, WB, ELISA; mouse, rat, human
https://www.ptgcn.com/products/PDCD6IP-Antibody-12422-1-AP.htm
CD9 Rabbit polyclonal antibodies Proteintech Cat# 20597-1-AP; FC, IHC, WB, ELISA; human, mouse, rat
https://www.ptgcn.com/products/CD9-Antibody-20597-1-AP.htm
CD63 Rabbit polyclonal antibody Proteintech Cat# 25682-1-AP; IF, IHC, WB, ELISA; human
https://www.ptgcn.com/products/CD63-Antibody-25682-1-AP.htm
CD63 (MX-49.129.5) mouse monoclonal antibodies Santa Cruz Cat# sc-5275; WB, IP, IF, IHC, IP, FCM, ELISA; mouse, rat, human
https://www.scbt.com/zh/p/cd63-antibody-mx-49-129-5?requestFrom=search
TSG101 Rabbit polyclonal antibody Proteintech Cat# 28283-1-AP; IHC, IP, WB, ELISA; human, mouse
https://www.ptgcn.com/products/TSG101-Antibody-28283-1-AP.htm
GOLGA2/GM130 Rabbit polyclonal Proteintech Cat# 11308-1-AP; FC, IF, IHC, WB, ELISA; human, canine
https://www.ptgcn.com/products/GOLGA2,GM130-Antibody-11308-1-AP.htm
Syntenin-1 Rabbit polyclonal antibody Proteintech Cat# 22399-1-AP; FC, IF, IHC, IP, WB, ELISA; human
https://www.ptgcn.com/products/SDCBP-Antibody-22399-1-AP.htm
ACTIN (2D4H5) Mouse monoclonal antibodies Proteintech Cat# 66009-1-Ig; FC, IF, IHC, IP, WB, ELISA; human, mouse, rat, hamster, monkey, dog, pig, chicken, rabbit, zebrafish
https://www.ptgcn.com/products/Pan-Actin-Antibody-66009-1-Ig.htm
HIF1a Rabbit polyclonal antibody Proteintech Cat# 20960-1-AP; FC, IF, IHC, IP, WB, ELISA; human
https://www.ptgcn.com/products/HIF1A-Antibody-20960-1-AP.htm
HIF1 alpha (AFfirm8002(AFB17813)) Mouse monoclonal Antibody Affinity Cat# BF8002; WB, IHC, IF/ICC; mouse
https://www.affbiotech.cn/goods-16579-BF8002-HIF1_alpha_Mouse_monoclonal_Antibody.html
Anti-UCP1 Abcam Cat# ab10983; WB, IHC, IP; mouse, rat
https://www.abcam.cn/ucp1-antibody-ab10983.html
Vinculin Rabbit mAb Abclonal Cat# A23468; WB, IHC, IP; human, mouse, rat
https://abclonal.com.cn/catalog/A23468
CREB (48H2) Rabbit mAb Cell Signaling Technology Cat# 9197;WB, IP, IHC, IF, FC, ChIP, C&R; human, mouse, rat, monkey, drosophila
https://www.cellsignal.cn/products/primary-antibodies/creb-48h2-rabbit-mab/9197?site-search-type=Products&N=4294956287&Ntt=9197&fromPage=plp&_requestid=3098024
Phospho-CREB (Ser133) (87G3) Rabbit mAb Cell Signaling Technology Cat# 9198;
WB, IHC, IF, FC, ChIP, C&R; human, mouse, rat
https://www.cellsignal.cn/products/primary-antibodies/phospho-creb-ser133-87g3-rabbit-mab/9198?site-search-type=Products&N=4294956287&Ntt=9198&fromPage=plp&_requestid=3100133
ATGL Antibody Cell Signaling Technology Cat# 2138; WB, IP, IF; human, mouse
https://www.cellsignal.cn/products/primary-antibodies/atgl-antibody/2138?site-search-type=Products&N=4294956287&Ntt=2138&fromPage=plp&_requestid=3100495
HSL Antibody Cell Signaling Technology Cat# 4107; WB, IP, IF; human, mouse
https://www.cellsignal.cn/products/primary-antibodies/hsl-antibody/4107?site-search-type=Products&N=4294956287&Ntt=4107&fromPage=plp&_requestid=3100652
Phospho-HSL (Ser563) Antibody Cell Signaling Technology Cat# 4139; WB; mouse
https://www.cellsignal.cn/products/primary-antibodies/phospho-hsl-ser563-antibody/4139?site-search-type=Products&N=4294956287&Ntt=4139&fromPage=plp&_requestid=3102740
Phospho-HSL (Ser660) Antibody Cell Signaling Technology Cat# 45804; WB; human, mouse
https://www.cellsignal.cn/products/primary-antibodies/phospho-hsl-ser660-antibody/45804?site-search-type=Products&N=4294956287&Ntt=45804&fromPage=plp&_requestid=3103130
VHL Antibody Affinity Cat# AF6292; WB, IHC; human, mouse, rat
https://www.affbiotech.cn/goods-1890-AF6292-VHL_Antibody.html
Leptin Antibody Affinity Cat# DF8583; WB, IHC, IF/ICC; human, mouse, rat
https://www.affbiotech.cn/goods-12056-DF8583-Leptin_Antibody.html
STAT3 Antibody Affinity Cat# AF6294; WB, IHC, IF/ICC; human, mouse, rat
https://www.affbiotech.cn/goods-1892-AF6294-STAT3_Antibody.html
Phospho-STAT3 (Tyr705) Antibody Affinity Cat# AF3293; WB, IHC, IF/ICC, IP; human, mouse, rat
https://www.affbiotech.cn/goods-1458-AF3293-Phospho_STAT3_Tyr705_Antibody.html
Ki67 Antibody Affinity Cat# AF0198; WB, IHC, IF/ICC; human, mouse, rat
https://www.affbiotech.cn/goods-897-AF0198-Ki67_Antibody.html
PE anti-human CD9 Antibody (HI9a) BioLegend Cat# 312106; FC; human
https://www.biolegend.com/en-us/products/pe-anti-human-cd9-antibody-2213

Eukaryotic cell lines

Policy information about cell lines and Sex and Gender in Research

Cell line source(s)

Cell lines used in this study were obtained from American Type Culture Collection and cultured in the recommended media or as indicated. These include MDA-MB-231 (HTB-26), 4T1 (CRL-2539), SK-BR-3 (HTB-30), MCF-7 (HTB-22), E0771 (CRL-3461), MCF-10A (CRL-10317), BT474 (HTB-20), 3T3-L1 (CL-173), HEK 293T (ACS-4500), NCM460 (CRL-9609), HCT116 (CCL-247), A549 (CCL-185), LLC (CRL-1642), NCM460 (CRL-9609), HCT116 (CCL-247), A549 (CCL-185). BEAS-2B was obtained from China Center for Type Culture Collection (GDC0139). C26 was gift from Dr. Pengcheng Bu of Institute of Biophysics， Chinese Academy of Sciences (Beijing, China). Both MGC-803 and GSE-1 cells were obtained from Dr. Wenhua Li of Wuhan University (Wuhan, China).

Authentication

Short tandem repeat profiling

Mycoplasma contamination	All cell lines were confirmed to be mycoplasma-free by PCR validation.
Commonly misidentified lines (See ICLAC register)	None

Animals and other research organisms

Policy information about studies involving animals; ARRIVE guidelines recommended for reporting animal research, and Sex and Gender in Research

Laboratory animals	Female NOD/SCID/IL2Rγ-null (NSG) mice (for EV tail vein injection, MDA-MB-231 xenograft model) were purchased from Shanghai Model Organisms Center, female BALB/c mice purchased from Center for Disease Control (CDC; Hubei, China) (for EV tail vein injection, 4T1 xenograft model), C57BL/6 mice were purchased from GemPharmatech (Nanjing, China) (for EV tail vein injection， LLC xenograft model), BKS-db mice （Strain NO.T002407） (for EV tail vein injection) were purchased from GemPharmatech (Nanjing, China).male BALB/c mice purchased from Center for Disease Control (CDC; Hubei, China) (for C26 xenograft model). Six to eight-week old female mice were used in all animal experiments. Mice were maintained in 12-hour light/dark cycles (6 am-6 pm) at 24°C with 50-60% humidity and fed standard irradiated rodent chow diet.
Wild animals	No wild animals were used in the study.
Reporting on sex	Male and female mice were used in this study.
Field-collected samples	No field collected samples were used in the study.
Ethics oversight	The animal protocols were approved by IACUC at Wuhan University. Tumors did not exceed the maximum volume of 1500 cubic mm.

Note that full information on the approval of the study protocol must also be provided in the manuscript.

Flow Cytometry

Plots

Confirm that:

☒ The axis labels state the marker and fluorochrome used (e.g. CD4-FITC).

☒ The axis scales are clearly visible. Include numbers along axes only for bottom left plot of group (a 'group' is an analysis of identical markers).

☒ All plots are contour plots with outliers or pseudocolor plots.

☒ A numerical value for number of cells or percentage (with statistics) is provided.

Methodology

Sample preparation	Extracellular vesicles (EVs) were purified by ultracentrifugation of conditioned medium as described in the method. Particle diameter and concentration were analyzed using flow cytometer (cytoFlexS, Beckman Coulter,Indianapolis,IN). Samples were stained with antibodies against CD9-labeled with phycoerythrin (PE,Biolegend) for 1h at RT, diluted 50-fold in buffer, and measured using membrane fluorescense to trigger detection. The analysis included calibration using calibrated microspheres with the size of 100 nm and 200 nm standard microspheres and a series of dilutions before staining to determine the optimal initial sample dilution and multiple positive and negative controls.
Instrument	CytoFlex, Beckman Coulter,Indianapolis,IN;Flow NanoAnalyzer, NanoFCM
Software	Cytexpert2.0 software;NF Profession2.0 software
Cell population abundance	EV concentrations are reported in EVs/ml,which is calculated from the number of events detected in the Vesicles geta and accounting for the volume analyzed and all pre- and post-stain dilutions. Marker-positive events we calculated from the number of events exceeding an arbitrary gate set at the ~99.5 percentile of the nagetive population.
Gating strategy	Data were analyzed using Cytexpert2.0 software. the first 20 seconds of data were discarded via a Time gate due to a consistent but unexplained background event anomaly observed on several different Cytoflex instruments. The remaining 105 seconds of data, corresponding to 100 ul of measured sample, and a plot of vFRed-A vs vFRed-H used to set a gate excluding certain background events that could be identified by their lower signal pulse area and widths. these events were further gated to include events with membrane fluorescence and light scatter intensity characteristic of EVs, and to exclude high light scatter intensity background events that have been noted in certain samples.

☒ Tick this box to confirm that a figure exemplifying the gating strategy is provided in the Supplementary Information.
